# Classification of CT scan and X-ray dataset based on deep learning and particle swarm optimization

**DOI:** 10.1371/journal.pone.0317450

**Published:** 2025-01-27

**Authors:** Honghua Liu, Mingwei Zhao, Chang She, Han Peng, Mailan Liu, Bo Li

**Affiliations:** 1 Hunan University of Chinese Medicine, Changsha, PR China; 2 Hunan University, Changsha, PR China; 3 Changsha Hospital of Traditional Chinese Medicine(Changsha Eighth Hospital), Changsha, PR China; 4 The First Hospital of Hunan University of Chinese Medicine, Changsha, PR China; Government College University Faisalabad, PAKISTAN

## Abstract

In 2019, the novel coronavirus swept the world, exposing the monitoring and early warning problems of the medical system. Computer-aided diagnosis models based on deep learning have good universality and can well alleviate these problems. However, traditional image processing methods may lead to high false positive rates, which is unacceptable in disease monitoring and early warning. This paper proposes a low false positive rate disease detection method based on COVID-19 lung images and establishes a two-stage optimization model. In the first stage, the model is trained using classical gradient descent, and relevant features are extracted; in the second stage, an objective function that minimizes the false positive rate is constructed to obtain a network model with high accuracy and low false positive rate. Therefore, the proposed method has the potential to effectively classify medical images. The proposed model was verified using a public COVID-19 radiology dataset and a public COVID-19 lung CT scan dataset. The results show that the model has made significant progress, with the false positive rate reduced to 11.3% and 7.5%, and the area under the ROC curve increased to 92.8% and 97.01%.

## Introduction

The COVID-19 pandemic has brought profound experiences and warnings to the world, significantly impacting our society, economy, and public health systems [1]. This pandemic has once again emphasized the importance of prevention and early response [2]. Early detection, rapid implementation of control measures, and strengthening of monitoring, early warning, and response mechanisms can greatly slow down the spread of the disease, reducing the number of cases and deaths.

Computed tomography (CT) is a frequently used medical imaging technique, which is widely used in clinical diagnosis and treatment. Meanwhile, X-ray is one of the commonly used methods of lung medical imaging, which can show abnormal shadows in the lungs. After the outbreak of the COVID-19 pandemic, these two methods have become an important tool for the diagnosis and assessment of COVID-19. These lung medical images have both high resolution and high contrast, allowing physicians to diagnose diseases more accurately, assess injuries, or plan surgeries.

Improving the accuracy of lung medical image classification undoubtedly enhances the detection and early warning capabilities of related diseases, thereby effectively suppressing the spread of the diseases. Traditional medical image classification methods usually depend on hand-designed feature extraction and machine learning algorithms. These methods require manually selecting and extracting features associated with a specific disease, and then using a classifier to perform the classification. However, because of the complexity and high dimensionality of lung medical images, traditional methods are often limited by the expressive and generalization capabilities of the features [3], which makes it difficult to capture the rich information in the images.

In recent years, the rapid development of deep learning technology has brought breakthroughs in medical image classification. Deep learning utilizes a multi-layer neural network model to achieve end-to-end image classification by learning and extracting features from a large amount of labeled data, which has a powerful nonlinear modeling capability and automatic learning ability and can effectively extract useful features from complex images to obtain accurate classification results [4]. In the clinical management and diagnosis of COVID-19, CT technology and X-ray technology have been widely used in the assessment of lung imaging in patients with suspected or confirmed infections. These medical images provide more intuitive and detailed information about the lung structure, which can help doctors determine the characteristics and extent of lung lesions and assist in the clinical diagnosis and therapeutic decision-making [5].

Due to the contagious and clinical characteristics of COVID-19, hospitals and healthcare organizations face the problem of large amounts of lung medical image data requiring rapid and accurate classification [6]. Accurately classifying COVID-19-infected medical images is crucial for early diagnosis, tracking disease progression, and guiding treatment. Therefore, deep learning-based lung image classification methods have become one of the hotspots of research. Deep learning methods automatically learn feature representations from a large amount of image data by constructing deep neural networks with strong nonlinear modeling and generalization capabilities and applying deep learning to COVID-19 lung image classification has become a promising solution [7]. In addition, medical image classification can help monitor outbreak transmission trends and assess epidemiological characteristics, providing necessary decision support for public health departments.

In recent years, there have been many studies on deep learning algorithms for CT images or Chest X-rays. The current approach primarily involves utilizing CNN networks to construct models for image classification or segmentation, such as ResNet, Inception, U-Net, and others. However, there are few studies on existing methods to reduce the false positive rate. They mostly focus on accuracy, AUC (Area Under the Curve) metrics, or F1-Score, while overlooking the FPR. Experiments have proved that over-pursuing AUC metrics in the training process will lead to a decrease in FPR, and experimental methods based on the data will consider each metric as equally important without weighting, and in the actual disease monitoring process, we are more accepting of the fact that negative patients being misclassified as positive, while positive patients being misclassified as negative will bring more losses and high FPR metrics can increase the cost of unnecessary testing and thus reduce the efficiency of disease detection.

In this paper, a low FPR method for disease detection based on COVID-19 lung images is proposed. The contributions are shown as follows.

1. Proposed a New Method for Disease detection: The paper introduces a novel method for detecting diseases, specifically COVID-19, using lung images. This method focuses on achieving a low false positive rate (FPR).
2. Integration of residual networks and particle swarm optimization: The proposed approach effectively combines residual networks and particle swarm optimization to minimize FPR while maintaining a satisfactory level of accuracy, measured by the Area Under the Curve (AUC) metrics.
3. Optimization of FPR in Model Training: The research addresses the challenge that FPR, typically used to evaluate model performance, is not a variable that can be directly optimized using traditional gradient descent methods.
4. Development of a Two-Stage Optimization Model. Stage 1: Utilizes classical gradient descent to train the model and extract relevant features. Stage 2: Constructs an objective function that minimizes FPR and employs an evolutionary algorithm to optimize model parameters, resulting in a network model with a low FPR.

The remainder of the paper is organized as follows: The Methods section explains the implementation of the low FPR neural network optimization model proposed in this paper, including the network structure and optimization algorithm;the Results and Discussion section explains the results of the comparative experiments and simulations done in this paper and finally the conclusions are given in the Conclusion section.

## Related work

Artificial intelligence-based Computer-Aided Diagnosis (CAD) is currently one of the most prominent research areas, playing a crucial role in diagnosing COVID-19 [8]. Many existing studies employ mainstream publicly available model architectures for CAD in lung medical imaging. For example, Jaiswal et al. [5] utilized DenseNet201 to classify lung CT images of COVID-19 patients. By employing transfer learning techniques to train the deep learning network, they achieved an accuracy of 96.29%. Similarly, Yu et al. [9] optimized the widely used GoogLeNet model to enhance its information extraction capability, demonstrating excellent performance on their private dataset. Fares Bougourzi et al. [10] introduced the PDAtt-Unet method, which achieved a specificity of 99.30%, precision of 74.74%, sensitivity of 80.69%, and an F1-Score of 77.60%. Furthermore, Ismael et al. [11] tested several pre-trained models, such as ResNet18, ResNet50, ResNet101, VGG16, and VGG19, on COVID-19 X-ray data, further confirming the effectiveness of deep learning techniques in COVID-19 diagnostic tasks.

In addition to adapting mainstream models, some researchers have proposed targeted model designs for COVID-19 lung imaging. Shah et al. [12] developed a custom CTnet-10 model, which required less training and prediction time compared to other publicly available network models, and achieved higher accuracy by extracting deep features from CT images. Attallah et al. [13] proposed a diagnostic approach that fused features from multiple CNNs to diagnose COVID-19, attaining high accuracy. Reshi et al. [14] aiming to address the imbalance and low quality of COVID-19 X-ray image datasets, introduced a deep CNN architecture that classified chest X-ray images and diagnosed COVID-19 with an impressive accuracy of 99.5% on an independent dataset.

Some researchers have improved accuracy by extracting deep features from data or optimizing various model parameters. For instance, Wang et al. [15] introduced a particle swarm optimization (PSO)-guided self-tuning CNN method. Similarly, Entesar et al. [16] employed optimization algorithms to fine-tune hyperparameters during model training. Both studies demonstrated that hyperparameter optimization not only reduced the time required for parameter adjustment but also enhanced model stability. Wang et al. [17] further combined feature extraction with parameter optimization. They first used wavelet Renyi entropy to extract deep features from images. These features were then fed into a neural network for learning, followed by the application of optimization algorithms to refine the network’s weight parameters and the Renyi entropy order.

Despite significant advancements, there is still room for improvement in optimizing model weight parameters. Many existing methods primarily focus on conventional metrics, such as overall accuracy, sensitivity, or specificity, to enhance model performance. However, relatively little research has been dedicated to optimizing specific metrics, such as the false-positive rate (FPR). In the context of disease detection, false positives can lead to additional medical testing, resource wastage, and increased unnecessary costs. To address this issue, this paper proposes a low-FPR method for COVID-19 prediction based on lung CT images.

## Methods

This study formulates disease detection as a binary classification problem and introduces a lightweight binary classification framework [18]. The proposed framework consists of two main components. The first component utilizes a deep neural network to extract discriminative features from three-channel two-dimensional image inputs. The second component leverages an evolutionary algorithm to optimize the model by freezing the parameters of other layers and updating only the weights of the fully connected layer. After the above two components, this paper builds a low FPR deep learning model.

### Proposed model

**Fig 1:**
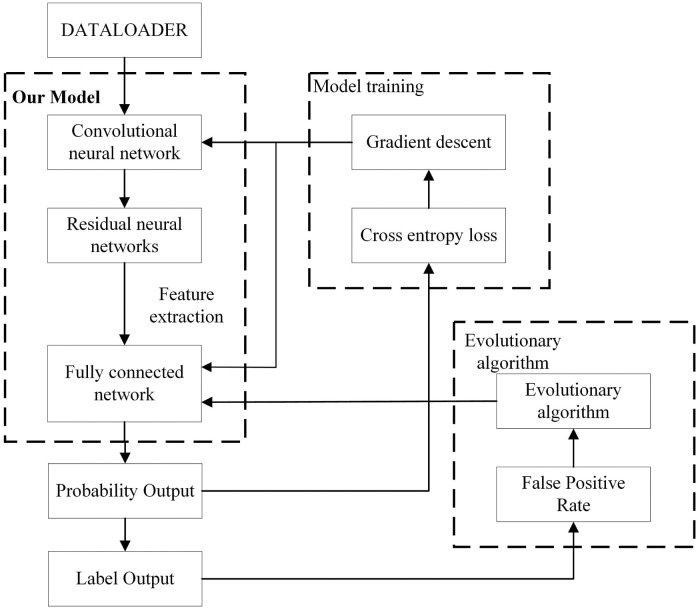
The framework of the proposed method.

**Fig 2:**
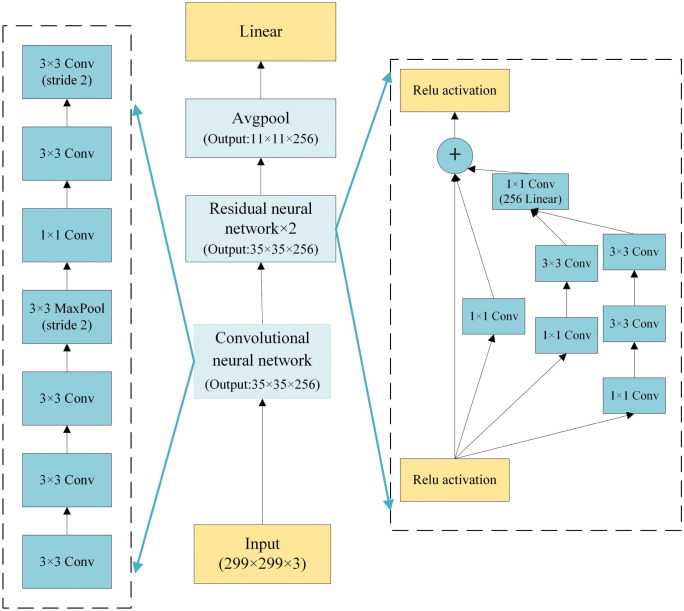
Layered architecture of model.

The low FPR network model proposed is mainly composed of the feature extraction network module and the optimization module, the structure is shown in Fig 1. The convolutional and residual layers first extract features from the image data to generate feature vectors. Then, the fully connected layer is used as the classification layer to output the probability distribution of classes, which is then translated into predicted labels. During the training process, the network weight parameters are updated using the gradient descent method. The convolutional and residual layers are then frozen, and the weights in the fully connected layer are fed into an evolutionary algorithm for training. Upon completion of training and updating of model weight parameters, classification results are output. Among them, the feature extraction network consists of a convolutional layer and a residual network layer, and the optimization module consists of an evolutionary algorithm and an FPR objective function, as shown in Fig 2. The convolutional layer consists of multiple 3 × 3 convolutional kernels and the number of kernels gradually increases as the number of layers increases, and the convolutional step size is 2 to reduce the size of the output feature map. At the same time, there is no dropout and pooling layer to maximize the data features that are passed to the residual network layer for feature extraction.

The residual network layer consists of several convolutional kernels of different dimensions 1×1, 3×3, and 5×5 to increase the adaptability of the network to different scales and to extract more global features. In this case, the 5×5 convolutional kernel is replaced by two 3×3 convolutional kernels to reduce computational complexity. Using convolutional kernels of different sizes allows for the extraction of richer features. A smaller kernel improves the efficiency of the network at capturing local features, while a larger kernel improves the performance of the network at capturing global features. At the same time, a 1×1 convolutional kernel is added before the convolutional kernel to downscale the input features to reduce the computational burden of larger convolutional kernels. At the same time, the inputs of each convolutional kernel are passed through a linear layer to combine the outputs of each convolutional kernel into a one-dimensional feature vector. Finally, residuals are computed with the input feature data to reduce network overfitting and solve the problems of gradient vanishing and gradient explosion. Finally, after a pooling layer with a kernel size of 3 × 3, the category of each feature is predicted by a fully connected layer, as shown in Fig 2. A dropout layer is added before the fully connected layer to reduce overfitting.

The feature extraction layer uses two convolution kernels of size 1 and 3. The residual layer uses a convolution layer with a convolution kernel size of 3, a stride of 1, and all-zero padding, and a convolution layer with a convolution kernel size of 1 and a stride of 1. Among them, the data normalization function BatchNorm2d uses default parameters, the batch size is 16, the initial learning rate is 0.001, and the number of training epochs is 100. The MultiStepLR function is used to dynamically adjust the learning rate. The number of adaptation epochs is 30. The optimizer uses AdamW, and the weight decay coefficient is 1e-6. The details of the architecture and hyperparameter values are given in Table 1.

**Table 1:** The details on and architecture and hyperparameter values.

Layer(type)	Output shape	Param
InputLayer	(3,299,299)	0
Features	(64,147,147)	28,928
MaxPooling2D	(64, 73, 73)	0
Features	(256, 35, 35)	624,368
ResidualUnit	(256, 35, 35)	42,432
ResidualUnit	(256, 35, 35)	42,432
AvePooling2D	(256, 11, 11)	0
Dropout	(256, 11, 11)	0
Linear	(None,None,1)	30977

### Training process of deep neural network

AUC, FPR, and TPR are three interrelated metrics, and typically, excessive focus on one metric will lead to changes in the other two metrics. In this paper, after extracting features using a deep learning network, we optimize the weight parameters of the fully connected layer of the network to reduce the FPR metrics, but excessive focus on the FPR metrics may lead to a decrease in AUC or TPR, so we should optimize these two interrelated metrics simultaneously to improve the AUC metrics and reduce the FPR as much as possible while ensuring a high degree of accuracy. Therefore, we design a multi-objective fitness function with linear weighting as the objective, and then we design a multi-objective fitness function as the objective. We designed a multi-objective fitness function with linear weighting as the objective function. In linear weighting, each evaluation metric is assigned a weight, and the sum of these weights is usually 1. The weights can be determined based on the importance of the task, the optimization objective, and the associated requirements. A higher weight indicates that the metric contributes more to the overall performance. The basic idea of the linear weighting method is to determine the indicators to be evaluated, AUC and FPR, and weight the indicators *ω*_1_, *ω*_2_, *ω*_1_, *ω*_2_ in the range of (0,1), where the ideal value of the FPR is 0%, and the ideal value of the AUC is 100%. The ideal value is 100%. Since both indicators can’t reach the ideal value, it is necessary to rationally evaluate the trade-off relationship between the indicators and introduce trade-off points to ensure that all indicators have effective changes, and the formula is expressed as follows:

$$\begin{array}{c} minFunc=\omega_{1}{\left(FPR-P_{1}\right)}^{2}+\omega_{2}{\left(AUC-P_{2}\right)}^{2}. \end{array}$$ (1)

where *ω*_1_ and *ω*_2_ denote the weights of each indicator, *P*_1_ denotes the ideal point for FPR and *P*_1_ should be close to 0, *P*_2_ denotes the ideal point for AUC and *P*_2_ should be close to 1.

Squaring each metric allows more attention to be paid to the optimization of a metric when the difference between that metric and the trade-off point is too large. In addition, to reduce the overfitting phenomenon, L2 regularization is introduced as a penalty term. The formula is expressed as follows:

$$\begin{array}{c} minFunc=Func\left(FPR,AUC\right)+\lambda \sum_{i=1}^{n}\omega_{i}^{2} \end{array}$$ (2)

### Optimize the FPR objective function

After training the network model in the first stage, we optimize the fully connected layer for the desired metrics, the algorithm used in this paper is the particle swarm optimization.

Particle Swarm Optimization (PSO) is a heuristic optimization algorithm inspired by the behavior of groups of organisms such as flocks of birds or schools of fish. It finds the optimal solution to a problem by modeling the collaboration and information exchange between individuals in a group [19]. In the particle swarm algorithm, the solution of the problem is represented as the position of a particle. Each particle has its position and velocity and searches based on the current position and velocity. The particle searches for the optimal solution by continuously updating its position and velocity. Meanwhile, the population search mechanism of the particle swarm algorithm converges faster, has strong robustness, requires fewer initial parameter settings, has a wide range of applicability, and the memory occupation is related to the number of particles and particle information.

The Particle Swarm Optimization is defined as:

$$\begin{array}{c} v_{id}^{k+1}=\omega_{1}v_{id}^{k}+c_{1}r_{1}\left(p_{id,pbest}^{k}-x_{id}^{k}\right)+c_{2}r_{2}\left(p_{id,gbest}^{k}-x_{id}^{k}\right) \end{array}$$ (3)

where, *v*_*id*_ denotes the velocity vector of the *i*_*th*_ particle, *x*_*id*_ denotes the position vector of the *i*_*th*_ particle, $p_{id,pbest}^{k}$ denotes the individual optimal solution, $p_{id,gbest}^{k}$ denotes the population optimal solution, *ω*_1_ denotes the inertia weight, *c*_1_ denotes the individual learning factor, *c*_2_ denotes the population learning factor, *r*_1_ and *r*_2_ denote random numbers within [0, 1].

The number of particles is chosen to be 30, the maximum number of iterations is set to 50, and the initial learning factors *c*_1_ and *c*_2_ are set to 1.5. The application of Particle Swarm Optimization is as follows.

- Initialize particle swarm: The weight parameters of the fully connected layers are aligned and merged into an array, with each parameter serving as the initial position and velocity of the particles.
- Compute fitness: For each particle, the fitness value, i.e., the objective function a, is computed based on its position function.
- Update individual optimal position: For each particle, update $p_{id,pbest}^{k}$, i.e. the optimal solution found so far by this particle, based on the current fitness value.
- Update optimal position of the population: Find the particle with the best fitness value in the population and use its position as the optimal position of the population $p_{id,gbest}^{k}$.
- Update velocity and position: For each particle, update the velocity and position of the particle according to $p_{id,pbest}^{k}\cdot$ and $p_{id,gbest}^{k}$, and the current velocity *v*_*i*_.
- Judge termination conditions: judge whether to terminate the algorithm based on predefined termination conditions (e.g., reaching the maximum number of iterations or meeting certain fitness requirements).
- Return result: the optimal solution found by using $p_{d,pbest}^{k}\cdot$ as the output of the algorithm and then loading the optimal solution into the original model to verify the validity of the algorithm.

When solving practical problems, it is normally required to search globally first and locally later, so it is necessary to adaptively change the size of the weights as the iteration proceeds, in this paper, we adopt the linear weighting method with the following formula:

$$\begin{array}{c} \omega =\omega_{\text{max}}-(\omega_{\text{max}}-\omega_{\text{min}})\frac{M}{M_{\text{max}}} \end{array}$$ (4)

### The flow of our algorithm

The flow steps of our algorithm are as follows.

- Step 1. Obtain image data from the original dataset.
- Step 2. Perform resizing, horizontal flipping, and vertical flipping on the images, followed by pixel normalization and data standardization to ensure adherence to a normal distribution.
- Step 3. Separate the data into training, validation, and test sets based on their respective categories.
- Step 4. Build the neural network model proposed in this paper.
- Step 5. Construct the training model based on the divided training data set, and evaluate the performance of the model using the validation data set at the end of each training epoch.
- Step 6. Freeze the parameters of the other layers and optimize the weights of the fully connected layer using the PSO optimization method proposed in the Optimizing the FPR Objective Function section.
- Step 7. Evaluate and test the trained and optimized model using the test set to verify the model’s performance.

## Results and discussion

This chapter first introduces the two datasets used in this study, followed by an explanation of the relevant metrics required for the comparative experiments. Finally, the experimental results are presented and discussed.

### Dataset selection and preparation

To verify the effectiveness of the proposed method, two datasets COVID-19 Radiography Dataset and the COVID-CT dataset were used to construct the experiment.

Dataset 1: COVID-19 Radiography Dataset, is comprised of 18,479 CXR images across 15,000 patient cases [20, 21]. The 925 of these cases are selected to construct the experiment, of which 427 are positive and 498 negative.

**Fig 3:**
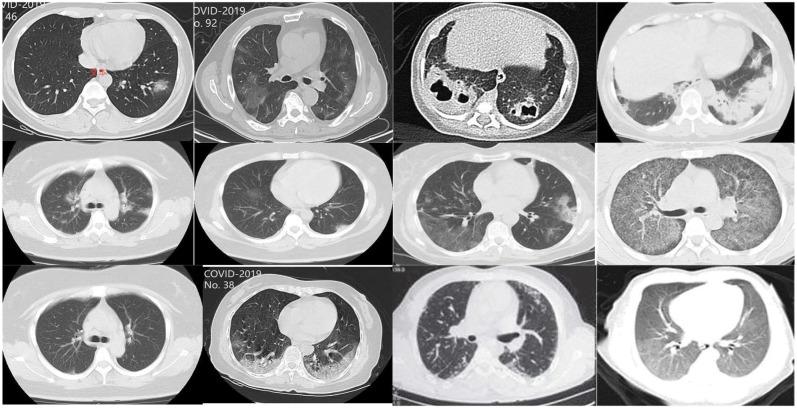
COVID-19 scan image dataset.

Dataset 2: The publicly available COVID-CT dataset consists of 812 CT scan images in total, including 349 images from COVID-19-positive patients and 463 images from COVID-19-negative patients [22]. As shown in Fig 3, a portion of the sample image from the dataset COVID-19 can be seen. Due to the different image quality of the acquired data, all images were transformed into RGB images of size (299, 299) to facilitate subsequent loading into the model for processing.

The two datasets, the open COVID-19 Radiography Dataset and the open COVID-19 lung CT scan dataset, utilized in the study are anonymized to ensure data security and individual privacy, encompassing sensitive information processing, encrypted storage and transfer, and compliance with privacy laws. Additionally, their long-standing public availability and usage in numerous papers underscore their transparency, validity, and representativeness, supporting the repeatability and verifiability of research findings. The data ratio is training set: validation set: test set = 7:1:2, and the data classification process uses stratified sampling and sets random seeds to ensure data balance. The details are shown in Table 2.

**Table 2:** Description of dataset.

	DATA1		DATA2	
Set	COVID-19	NON-COVID-19	COVID-19	NON-COVID-19
Train	299	349	244	278
Val	43	49	35	40
Test	85	100	70	79
Total	427	498	349	397

### Evaluation metrics

Appropriate evaluation metrics are the key to evaluating the performance of a model. At present, most experimental methods choose traditional confusion matrix calculation metrics, such as accuracy, precision, AUC, F1-Score, and other metrics, which evaluate the overall performance of the model, and in practical medical image processing tasks, more attention is paid to evaluating the AUC metric while ignoring the FPR. In the field of disease monitoring and medical diagnosis, misclassifying a sick patient as a healthy negative patient compared to misclassifying a healthy positive person as sick can lead to greater losses and increase unnecessary testing and treatment costs. AUC measures the classification ability of a model under different thresholds, of which FPR is a component, and optimizing the area under the curve (AUC) is a common goal of model training; a higher AUC value indicates that the model has better predictive ability under different classification thresholds and can more accurately distinguish between positive and negative situations. However, too much focus on improving the AUC can lead to some problems. A common scenario is that the model may tend to predict samples as negative cases to reduce the number of false positives and thus lower the FPR. In this case, the model may produce some compression in the prediction of positive case samples, which may cause some true positive cases to be incorrectly predicted as negative cases. The model can improve the prediction accuracy of negative example samples and reduce the misclassification of negative examples as positive examples. At the same time, however, it may cause some true positives to be misclassified as negatives, increasing the risk of false negatives.

Balancing the risk of false positives and negatives is a critical consideration in the model optimization process. Selecting appropriate classification thresholds and employing appropriate strategies can help address this issue. At the same time, combining the knowledge and experience of domain experts with an understanding of specific application scenarios can better balance and manage the predictive accuracy and risk of the model. For example, in domains such as disease surveillance, focusing only on AUC improvement during training may not be the best strategy. Different evaluation metrics need to be considered comprehensively, and appropriate optimization goals and evaluation metrics need to be determined by weighing them according to the actual needs and application scenarios. Since the negative patient label is 1 and the positive patient label is 0 when the dataset is divided, to pay more attention to the misclassification rate of the positive patients, the method in this paper selects the indexes of AUC and FPR and optimizes the network model to reduce the FPR index while maintaining a certain AUC value.

- AUC: AUC is one of the commonly used metrics for evaluating the performance of the binary classification model [23]. The ROC curve (Receiver Operating Characteristic) is plotted with the True Positive Rate (TPR) (also known as recall or sensitivity) at different thresholds as the horizontal coordinate, and False Positive Rate (FPR) as the vertical coordinate. AUC represents the area under the ROC curve, which ranges from 0 to 1. The closer the AUC is to 1, the better the model is at different thresholds. Positive Rate (FPR) as the vertical coordinate. AUC denotes the area under the ROC curve, which ranges from 0 to 1. The closer the AUC is to 1, the better classification ability the model has under different thresholds.
- FPR: FPR is the percentage of samples in which the model incorrectly predicts a negative case as a positive case. In image processing tasks, especially in some applications with high sensitivity (e.g., medical imaging), it is important to reduce the FPR. By focusing on the FPR, it is possible to ensure that the model’s false alarm rate in negative case samples is as low as possible, thus increasing the model’s reliability and practical application value. In this experiment, the prevalence data is set to label 0, so the FPR is defined in this paper as the proportion of patients with COVID who are incorrectly diagnosed as not having COVID. The FPR is defined as:
  $$\begin{array}{c} FPR=\frac{FP}{FP+TN} \end{array}$$ (5)
- F1-Score: The F1-Score score is a crucial metric for assessing the effectiveness of a binary classification model. It considers the model’s proficiency in detecting both positive and negative examples. Detecting positive examples involves correctly identifying the number of genuinely positive samples, while detecting negative examples involves accurately recognizing the number of truly negative samples.
  $$\begin{array}{c} F1\text{-Score}=\frac{2*TP}{2*TP+FP+FN} \end{array}$$ (6)
  where FP denotes false positive, TN denotes true negative, TP denotes true positive, and FN denotes false negative.
- PREC: Precision is the proportion of samples predicted to be positive that are actually positive. It measures the reliability of the model’s predictions.
  $$\begin{array}{c} \text{PREC}=\frac{TP}{\left(TP+FP\right)} \end{array}$$ (7)
- SPEC: Specificity refers to the proportion of samples predicted to be negative by the model that are truly negative. It measures the ability of the model to distinguish between true negatives.
  $$\begin{array}{c} \text{SPEC}=\frac{TN}{\left(\begin{array}{c} TN+FP \end{array}\right)} \end{array}$$ (8)

### Experimental result

The dataset is manually divided into training, test, and validation sets in the ratio of 7:2:1, and one hundred training rounds are performed, with each round using the test set data to validate the model results, and finally, plots of the accuracy analysis results of the validation and test sets for each model are generated. The deep learning model proposed in this paper and the deep learning model involved in the experimental comparison are implemented using the open-source deep learning framework of Pytorch, which runs under the environment of python3.7.

**Fig 4:**
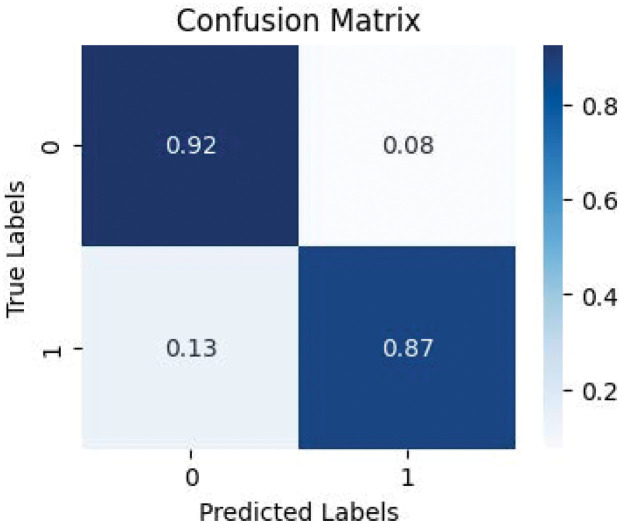
Confusion matrix of our method in dataset 1 and dataset 2.

The confusion matrix is shown in Fig 4. The confusion matrix is an essential tool in classification models, providing a visual representation of an algorithm’s performance. It consists of four elements: True Positives (TP), which indicate the correct identification of a disease; False Positives (FP), which indicate the incorrect identification of a non-existent disease; True Negatives (TN), which confirm the correct non-existence of a disease; and False Negatives (FN), where the model fails to identify an existing disease. These components allow us to calculate important metrics such as accuracy, precision, recall, and F1-Score. As shown in the matrix, the results indicate an excellent detection capability with an accuracy rate of over 85%.

**Fig 5:**
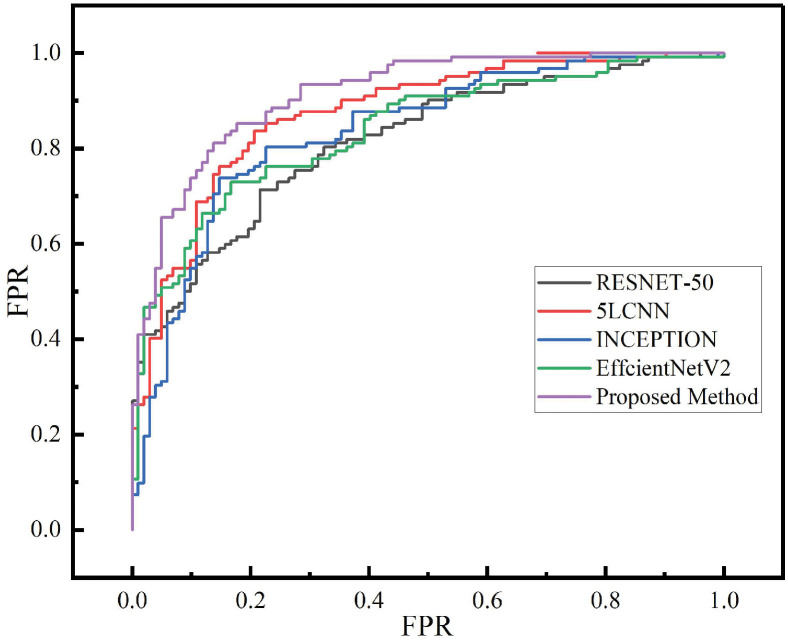
ROC curve among different methods in dataset 1 and dataset 2.

The proposed network model is used to validate the model on the COVID-19 radiography dataset and COVID-19 dataset, and then Lightweight Multi-scale CNN (LIMS) [24] and hybrid deep learning models(HDLM) [25], RESNET-50 [26], INCEPTION [27], a five-layer deep convolutional neural network (5LCNN) [28], EfficientNetV2 [29] are used for comparative experiments. RESNET-50, with its 50 layers, enables efficient performance of tasks such as image classification using skip connections. INCEPTION uniquely combines convolutions of different sizes for cost-effective parameter use, inspired by the retina. The 5LCNN introduces the method of stochastic pooling, providing robustness against overfitting and computational efficiency. EfficientNetV2, part of the Google family of models, introduces scalable improvements that address speed and accuracy, optimizing performance across devices. The LIMS has a special network model architecture, and the Hybrid Deep Learning model is a converged network using VGG-19 and VGG-16. The experimental results are shown in the following two tables, Tables 3 and 4. The ROC curve between different methods in data set 1 and data set 2 is shown in Fig 5.

**Table 3:** Analysis of the proposed model in the COVID-19 radiography dataset.

Metric	ACC(%)	AUC(%)	FPR(%)	F1-Score(%)	PREC(%)	SPEC(%)	Time (s)	p-value
Model								
RESNET-50	87.05	96.29	12.78	87.50	88.11	87.22	0.1990	<0.05
INCEPTION	78.06	87.34	24.06	79.18	78.38	75.94	0.0467	<0.05
5LCNN	89.57	94.89	12.03	90.10	89.19	87.97	0.0760	0.1
EfficientNetV2	83.81	91.99	14.29	84.10	86.23	85.71	0.8700	<0.05
LMIS	89.57	95.97	17.29	**90.55**	85.80	82.71	**0.0314**	0.1
HDLM	87.14	94.02	15.79	88.22	86.18	84.21	0.5090	<0.05
Proposed Method	**89.57**±3.59	**97.01**±2.0	**7.52**±3.1	89.68±3.58	**92.65**±3.07	**92.48**±3.1	0.411	-

**Table 4:** Analysis of the proposed model in the COVID-CT dataset.

Metric	ACC(%)	AUC(%)	FPR(%)	F1-Score(%)	PREC(%)	SPEC(%)	p-value
Model							
RESNET-50	73.66	81.50	26.47	75.31	76.92	73.53	<0.05
INCEPTION	75.45	83.80	23.53	76.79	79.13	76.47	<0.05
5LCNN	80.36	87.36	20.59	81.82	82.50	79.41	<0.05
EfficientNetV2	74.11	84.02	15.69	73.39	83.33	84.31	<0.05
LMIS	79.46	88.20	23.53	81.30	80.65	76.47	<0.05
HDLM	79.91	86.31	19.61	81.17	82.91	80.39	<0.05
Proposed Method	**83.04**±4.91	**91.37**±3.68	**12.75**±4.37	**83.62**±4.85	**88.18**±4.23	**87.25**±4.37	-

In the performance assessments conducted on two distinct datasets, COVID-19 Radiography Dataset (Dataset 1) and COVID-CT dataset (Dataset 2), the proposed method outperformed well-established methodologies such as ResNet-50, Inception, 5LCNN, EfficientNetV2, LIMS and Hybrid Deep Learning Model across the majority of evaluation metrics, including accuracy, area under the ROC curve, false positive rate, precision, and specificity. The only deviation was a slightly lower F1-Score than the 5LCNN and LIMS in Dataset 1, which results in insignificant significant differences in p-value. Nonetheless, in Dataset 2, the proposed method excelled in all six evaluation metrics, outshining the four comparative approaches. These results unequivocally validate the effectiveness of the proposed method in COVID-19 detection imaging tasks and its exceptional and consistent performance across diverse datasets.

To verify the effectiveness of different steps of the method, we constructed ablation experiments, the proposed method without Residual unit (Without RES), the proposed method without PSO (Without PSO), and the proposed method. The analysis of the proposed model in dataset1 and dataset2 in Tables 5 and 6.

**Table 5:** Ablation experiments in dataset 1.

COVID-19 Radiography Dataset						
	ACC	AUC	FPR	F1-Score	PREC	SPEC
Without RES	0.8921	0.9658	0.0977	0.8951	0.9078	0.9023
Without PSO	0.8885	0.9632	0.1504	0.8963	0.8701	0.8496
Proposed Method	**0.8957**	**0.9701**	**0.0752**	0.8968	**0.9265**	**0.9248**

**Table 6:** Ablation experiments in dataset 2.

COVID-CT dataset						
	ACC	AUC	FPR	F1-Score	PREC	SPEC
Without RES	0.808	0.8879	0.2157	0.8245	0.8211	0.7843
Without PSO	0.8125	0.8914	0.1667	0.822	0.8509	0.8333
Proposed Method	**0.8304**	**0.9137**	**0.1275**	**0.8362**	**0.8818**	**0.8725**

The ablation studies highlighted in Tables 5 and 6, using the COVID-19 Radiography Dataset and the COVID-CT dataset, reveal that our method outshines other variations, enhancing key performance metrics. Specifically, the omission of residual modules and Particle Swarm Optimization resulted in a decrease in model accuracy (ACC), area under the ROC curve (AUC), false positive rate (FPR) by nearly 23%, F1-Score, precision (PREC), and specificity (SPEC) in the Radiography Dataset. Implementing our full method not only recovered but also improved these metrics. This pattern of declination and subsequent improvement with the complete method was also noted in the COVID-CT dataset performance, emphasizing the essential role of the included components in refining the model’s effectiveness.

### Limitation and discussion

The remarkable results achieved in Tables 3 and 4 can be attributed to three distinctive steps incorporated in the proposed method. First, multilayer convolution is used to efficiently extract image features. This technique captures an array of features at different depths, providing the model with enhanced representational capacity. Next, the architecture integrates residual modules that mitigate the gradient vanishing problem prevalent in conventional deep networks, allowing for more effective training and improved model performance. Finally, Particle Swarm Optimization (PSO) is used to fine-tune the false positive rate (FPR). PSO, an algorithm rooted in swarm intelligence, quickly and accurately refines the global optimum for the optimization problem at hand. Through this optimization process, the model skillfully minimizes the false positive rate, significantly reducing false alarms while maintaining a high detection rate, and increasing the practical applicability and value of the model.

The time spent of these methods is shown in Table 3. The results show that the time used by our method is less than 0.50 seconds, which is close to the minimum time consumption and far less than the time consumption of other methods.

The remarkable results obtained in Tables 3 and 4 show that on the COVID-19 radiography and COVID-CT datasets, the removal of residual modules (without RES) and Particle Swarm Optimization (without PSO) degrade performance metrics such as ACC, AUC, FPR, F1-Score, PREC, and SPEC; whereas the full implementation of the proposed method significantly improves the results, in particular reducing FPR by almost 23%, highlighting its effectiveness in reducing false positives.

The results of evaluating the model on two data sets (Data Set 1 and Data Set 2) show the values for various performance metrics along with the confidence intervals at the 95% confidence level. For Data Set 1, the precision of the model is 0.8957 with a confidence interval of [0.8598, 0.9316], and there are corresponding values and confidence intervals for metrics such as AUC, FPR, F1-Score, precision, and specificity. For Data Set 2, the confidence interval for precision is [0.7813, 0.8795], AUC is 0.9137 with a confidence interval of [0.8769, 0.9505], and there are corresponding values and confidence intervals for FPR, F1-Score, precision, and specificity.

The impact of data preprocessing/augmentation techniques on model performance can vary depending on the specific model and task. It is important to use the same data preprocessing/augmentation techniques for training and testing data. Data preprocessing/augmentation techniques can be computationally expensive, so it is important to choose effective techniques. The performance of machine learning models can be improved by carefully considering the data preprocessing/augmentation techniques used. To compare the performance of the model itself, the proposed method uses only simple data processing methods. First, the image is flipped horizontally and vertically, then the pixel values of the image are normalized, and the data is standardized to follow a normal distribution. This process reduces the cost of data augmentation and the computational cost of the model, which is more conducive to the later use of the model.

Although the proposed method works extremely well in Data Set 1 and Data Set 2, it is not a substitute for clinical trial results. The results of the model can support clinical trials by providing physicians with a diagnostic reference based on patient symptoms, identifying high-risk individuals, and allowing early intervention to reduce disease incidence. It can also streamline patient care by quickly screening low-risk cases and reducing waiting times, but the model results can only be used as a reference in a limited number of situations and are not a substitute for professional medical judgment.

## Conclusion

In this paper, we design a low FPR method for disease prediction based on COVID-19 lung images. The ACC and AUC in the validation set of the COVID-19 chest CT scan data set are 83.04% and 91.37%, and the accuracy and AUC of the COVID-19 chest X-ray data validation set are 89.57% and 97.01%, respectively, which are much higher than those of other methods, including some recent methods. The FPR metrics of the low-FPR deep neural network model designed in this paper are 12.75% and 7.52% in the CT and chest X-ray data sets, respectively. This indicates that the proposed method is superior to the corresponding metrics of other comparative models, and has a more obvious improvement compared to the metrics before optimization.

In clinical practice, failure to detect positive cases is often considered unacceptable. The method proposed in this study improves the accuracy of positive case detection by optimizing the FPR, thereby increasing the model’s sensitivity to positive cases and improving the predictive accuracy and reliability, while the two-stage model optimization approach can further improve the model’s performance within defined constraints. In the future, we will collect a large number of medical imaging datasets for different pathologies and use these data to further validate and test the model. By testing the model under different pathological conditions, we will identify the model’s limitations and areas for improvement, and then further optimize and improve the existing model to increase its versatility in different diseases.

This study primarily addresses the binary classification problem between diseased and normal samples, without addressing the classification of different disease types and their pathological features. However, in actual clinical practice, raw image data often contain multiple disease categories and are typically affected by problems such as data imbalance and poor quality. Therefore, future research should focus on the following directions while continuing to optimize model performance and metrics: Designing loss functions suitable for multi-class tasks to address the problem of class imbalance; Applying data augmentation techniques to improve the model’s robustness to sample variation; Using transfer learning to learn generalizable features from large medical imaging datasets, thereby improving the model’s ability to generalize in new environments. In addition, given the difficulty of fully adapting deep learning models to the complex and variable nature of clinical applications, it is necessary to establish dynamic updating and continuous learning mechanisms.

In summary, in addition to optimizing model performance, it is also necessary to explore diversified solutions for data quality, category imbalance, model generalization, etc., and build an AI diagnostic system with dynamic updates and continuous learning to ensure its long-term applicability in complex medical environments.

## Supporting information

S1 File (ZIP)
